# Kidney Biopsy and Type IV Collagen Gene Sequencing Fail to Explain Hematuria in Loin Pain Hematuria Syndrome

**DOI:** 10.1016/j.ekir.2023.02.1075

**Published:** 2023-02-22

**Authors:** Bhanu Prasad, Aditi Sharma, Mathew B Lanktree, Kunal Goyal, Pouneh Dokouhaki

**Affiliations:** 1 Section of Nephrology, Department of Medicine, Regina General Hospital, Regina, Saskatchewan, Canada; 2 College of Medicine, University of Saskatchewan, Regina, Saskatchewan, Canada; 3 Institute for Microbial Systems and Society, University of Regina, Regina, Saskatchewan, Canada; 4 Division of Nephrology, St. Joseph’s Healthcare Hamilton, McMaster University, Hamilton, Ontario, Canada; 5 Department of Radiology, Regina General Hospital, Regina, Saskatchewan, Canada; 6 Department of Pathology and Laboratory Medicine, University of Saskatchewan, Saskatoon, Saskatchewan, Canada

**Keywords:** glomerular basement membrane, kidney biopsy, loin pain hematuria syndrome, rare disease, thin basement membrane disease, type IV collagen

## Abstract

**Introduction:**

Loin pain hematuria syndrome (LPHS) is a rare clinical syndrome with a reported prevalence of 1 in 10,000. The syndrome is characterized by severe pain localized to the kidney in the absence of identifiable urinary tract disease. Because of an inadequate understanding of the pathophysiology of the disease, the goal of management has been limited to symptomatic pain management. Through detailed phenotype and genotype assessment we sought to identify possible underlying etiologies.

**Methods:**

We completed a chart review, ultrasound imaging, kidney biopsy, and type IV collagen (*COL4A3*, *COL4A4*, and *COL4A5*) gene sequencing in 14 patients with loin pain hematuria recruited from a single center.

**Results:**

Red blood cells and red cell casts were observed within the tubules in 10 of 14 patients. The glomerular basement membrane (GBM) was normal in 11 patients and thickened in 1 patient. Staining for IgA kappa was present in 1 patient. C3 deposition without any inflammation was present in 7 patients. Arteriolar hyalinosis was present in 4 patients and endothelial cell injury was present in 6 patients. No pathogenic *COL4A3*, *COL4A4*, or *COL4A5* variants were identified.

**Conclusion:**

Conventional histopathology and genetic testing for type IV collagen variants failed to identify the cause of hematuria in 14 patients with LPHS.

## Graphical abstract

**Figure:**
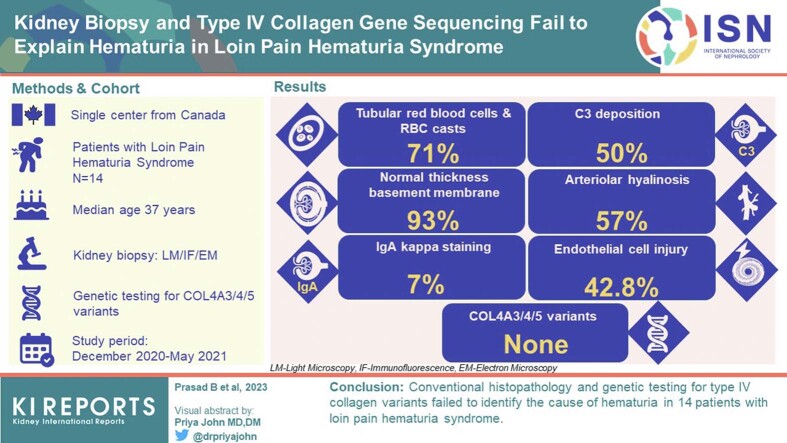

Since its first description by Little and colleagues in 1967, the etiology of LPHS remains poorly understood.^1^ The diagnosis is made in the presence of fluctuating but chronic, severe unilateral, or bilateral flank pain accompanied by either microscopic or gross hematuria and after exclusion of urological sources of pain and hematuria such as kidney stones and infection.^2^ LPHS remains a rare clinical entity with prevalence estimates in the range of 1 in 10,000.^2^ However, for those affected, symptoms severely impact daily functioning and quality of life. Kidney function, as evaluated with estimated glomerular filtration rate, is typically maintained and there is a lack of associated proteinuria. The gold standard to evaluate isolated hematuria is a thorough evaluation of the GBM on a kidney biopsy. However, nephrologists have been reluctant to perform kidney biopsies given preserved estimated glomerular filtration rate and lack of proteinuria.^3^ The lack of a pathophysiological understanding has made targeted therapies impossible and has left patients without a concrete diagnosis. We sought to perform a histopathological examination of kidney biopsy samples and sequence type IV collagen genes to see if it improved diagnostic precision in patients with LPHS.

For glomerular hematuria to occur, red blood cells need to pass a 3-layered barrier consisting of glomerular endothelial cells, GBM, and podocytes.^4^ Podocyte injury typically results in proteinuria, which is absent in LPHS. Isolated hematuria is frequently associated with injury to the GBM, whereas the functional importance of endothelial cells in hematuria remains unclear. Type IV collagen is the major collagen constituent of the GBM. Rare pathogenic variants in the 3 genes that code for type IV collagen (*COL4A3, COL4A4,* and *COL4A5*) have increasingly been recognized as common causes of glomerular hematuria. Hemizygous severe pathogenic variants in *COL4A5* lead to classical Alport syndrome in boys and young men with hematuria, proteinuria, and rapid decline in estimated glomerular filtration rate as well as extrarenal manifestations of ocular and hearing abnormalities. Nevertheless, the population prevalence of heterozygous type IV collagen variants was about 1 in 1000 in the UK biobank, which is 5 to 16 times more frequent than historic prevalence estimates of Alport syndrome.^5^

The mechanism of pain in LPHS is also unclear. There is a consensus that the hematuria is glomerular in origin but it remains uncertain if the pain is interrelated with the hematuria. In 1996, Hebert *et al.*^6^ proposed glomerular hematuria as the instigating event, sequentially leading to tubular obstruction, back leak of glomerular filtrate, and local parenchymal edema that promotes compression of adjacent tubules and subsequent capsular stretch leading to pain. Challenging the above stated mechanism is the absence of pain associated with other types of glomerular hematuria, such as seen in most cases of nephritic glomerulonephritis. Only 2 small studies, consisting of fewer than 50 patients in total, have examined the pathophysiology of LPHS*.*^7^^,^^8^ Previous biopsy studies looking at glomerular causes of hematuria have failed to reveal the source of hematuria in a consistent manner with results varying from no structural changes to presence of IgA^8^^,^^9^ and thin basement membrane nephropathy.^6^^,^^10^ No previous studies have evaluated a potential co-occurrence of pathogenic type IV collagen variants and LPHS.

Because there are gaps in our understanding, we sought to assess the histopathological features on kidney biopsy in 14 patients with loin pain hematuria, and specifically, to evaluate the correlation of GBM alterations by electron microscopy with hematuria. Further, in keeping with the recent advances in our understanding of type IV collagen’s role in hematuria, we performed targeted sequencing of *COL4A3*, *COL4A4*, and *COL4A5*.

## Methods

### Patient Population

**Figure 1:**
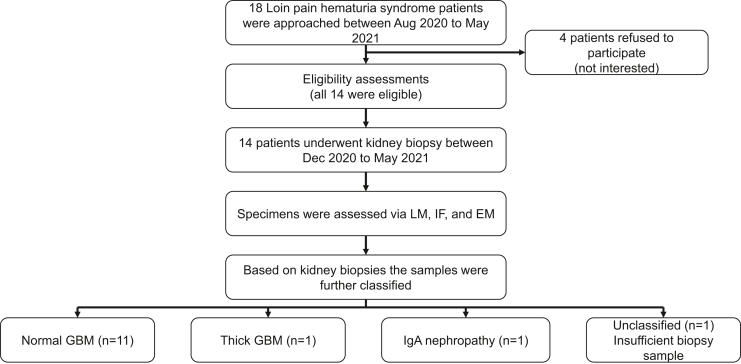
Flow diagram. EM, electron microscopy; GBM, glomerular basement membrane; IF, immunofluorescence.

Eighteen consecutive patients referred to nephrology clinic (run by the corresponding author) were approached by a study coordinator and 14 agreed to participate in the study (Figure 1). Because of our interest in LPHS, we receive referrals from all across Canada. Consistent with previous literature, we required the following conditions to be met: (i) pain characteristic of kidney pain; (ii) chronic fluctuating pain requiring prescription opiates; (iii) gross or microscopic hematuria (≥5 red cells per high power field) on repeated urine microscopy; and (iv) if urolithiasis had occurred in the past, absence of obstruction was confirmed by imaging during the episode of pain.^2^ Patients were diagnosed with LPHS by a nephrologist (BP), in consultation with a urologist (FG). The enrolled patients signed a written consent form before participation. The study was approved by the Saskatchewan Health Authority Research Ethics Board (REB 18-29).

Demographic data, including sex, age, and self-reported ethnicity, was gathered. Phenotype information including age of onset of symptoms, comorbidities (diabetes, hypertension, or previous kidney stone), weight, height, smoking status, and current medications was reported by patients. Morphine milligram (mg) equivalent per day was determined by using an equivalency factor to calculate a dose of morphine that is equivalent to the used opioid. Family history of LPHS, kidney stones, chronic kidney disease or end-stage kidney disease, or chronic pain was collected.

After enrollment, serum creatinine and electrolytes; complete blood count; and urine dip, microscopy, and albumin-to-creatinine ratio were measured. All patients underwent a cystoscopy, ultrasound assessment of the kidneys and urinary tract, triphasic computed tomography of the abdomen and pelvis with delayed images, and split function mercaptoacetyltriglycine scan to assess the relative function of the kidneys. All patients had a negative urological assessment for causes of pain and hematuria (obstructive stones, cysts, malignancy, thromboembolism, renal vein entrapment syndrome, trauma, or papillary necrosis).

### Kidney Biopsy

Patients underwent kidney biopsies from December 2020 to May 2021 at the Regina General Hospital, Saskatchewan, Canada. Kidney biopsies were performed by an interventional radiologist under realtime ultrasound guidance. Three cores were taken with a spring-loaded biopsy gun using an 18-gauge needle (Argon Medical Devices Inc, Athens, TX, USA). All biopsies were read by a single kidney pathologist (PD) at the Department of Pathology and Laboratory Medicine, University of Saskatchewan, Saskatoon, Saskatchewan, Canada. The samples obtained were divided into 3 portions for light microscopy, immunofluorescence, and electron microscopy (EM). The sample for light microscopy was fixed in formalin and embedded in a paraffin block. Sections of 1 to 3 mm thickness were cut and stained with hematoxylin and eosin, periodic acid–Schiff, Masson trichrome, and periodic acid silver methenamine special stains. The prepared slides were assessed using Nikon Eclipse Ci (Nikon, Japan) microscope, using magnifications from 40 to 600 times. Each case was assessed and scored for global and segmental glomerulosclerosis, tubular injury, interstitial fibrosis and tubular atrophy, arteriosclerosis, arterial intimal fibrosis, and presence of red blood cells or degenerated red cell casts in tubules.

Direct immunofluorescence was performed by adding fluorescein-conjugated antibodies against immunoglobulin heavy chains (IgG, IgA, IgM), light chains (kappa and lambda), complement components (C1q, C3), and albumin using Benchmark ULTR system from Roche Diagnostics following the manufacturer’s instructions. Samples were then examined under blue laser light using an immunofluorescent microscope.

Ultrathin sections were stained with uranyl acetate and lead citrate before examination under the transmission electron microscope (Hitachi HT7800, Japan). GBM thickness was measured digitally using an EM image analysis system after calibration. Measurements were obtained in properly oriented open peripheral glomerular capillaries, with a minimum of 3 measurements evenly distributed per loop. GBM measurements were obtained in a minimum of 10 glomerular capillary loops of at least 2 viable glomeruli when possible. Mean and range of GBM thickness were then calculated and recorded for each sample. For standardization, mean and range of GBM thickness were reported for each sample, as previously reported.^11^ The GBM was classified as “thick” if the average width was >395 nm and “thin” if membrane thickness was <250 nm.^11^ This cut off is established based on 2 SDs below the average for normal GBM thickness in women as reported by Steffes *et al.*^12^

### Genetic Testing

Genetic testing was performed at Clinical Laboratory Improvement Act certified clinical laboratory approved by Saskatchewan Health Authority. DNA was extracted from leukocytes obtained from whole blood specimens. Sequencing included coding regions and 10 bases of exon flanking region. DNA corresponding to *COL4A3, COL4A4,* and *COL4A5* was captured using hybridization probes. Captured DNA was pair end sequenced (150 × 2) on NovaSeq6000 using Illumina’s reversible dye terminator technology. The Clinical Laboratory Improvement Act certified laboratory used the following quality control metrics: >98% of targeted bases covered at >20× and mean coverage of target bases >100×. Copy number evaluation defined as single exon or larger deletions or duplications (Del/Dups) were detected from the sequencing data by the Clinical Laboratory Improvement Act laboratory using their proprietary bioinformatics pipeline. All sequencing results were reviewed and interpreted by clinical molecular geneticists. All sequencing results were reviewed and interpreted by clinical molecular geneticists. Sequence variants were categorized as “pathogenic”, “likely pathogenic”, “benign”, “likely benign”, and “variant of uncertain significance” as per American College of Medical Genetics Guidelines.^13^

### Statistical Analysis

Descriptive statistics were used to report baseline characteristics and histology findings. Results were expressed as count (%) for categorical variables and median (interquartile range) for continuous variables.

## Results

Among 18 patients who were approached, 14 agreed to participate and their baseline characteristics are in Table 1. All patients were female, with median age of 37 years (interquartile range, 33.5–43.3). Most of the patients (11 of 14, 78%) were of self-reported European ancestry. Eight patients (57%) had a preceding history of kidney stones but none had an obstructing stone on imaging at the time of continued loin pain. Eleven patients (79%) had episodes of macroscopic hematuria, with 7 reporting gross hematuria with exacerbations of loin pain. Ten patients (71%) had unilateral pain, with chronic pain for a median duration of 6 years. None of the patients had evidence of chronic kidney disease with no patients with an abnormal estimated glomerular filtration rate or an elevated albumin-to-creatinine ratio (>3 mg/mmol). The median creatinine was 64 μmol/l (interquartile range, 54.7–69.2).

**Table 1:** Cohort characteristics

Baseline characteristics	Total cohort (*N* = 14)	Primary loin pain hematuria syndrome		Secondary loin pain hematuria syndrome
		Normal *(n =* 11)	Thick (*n* = 1)	IgA (*n* = 1)
Age (yr), median (IQR)	37 (34–43)	37 (32–44)	42	35
Sex (female), *n* (%)	14/14 (100%)	11/11 (100%)	1/1 (100%)	1/1 (100%)
Self-reported ethnicity				
European, *n* (%)	11/14 (79%)	9/11 (82%)	1/1 (100%)	1/1 (100%)
Aboriginal, *n* (%)	2/14 (14%)	1/11 (9%)	0/1 (0%)	0/1 (0%)
South Asian, *n* (%)	1/14 (7%)	1/11 (9%)	0/1 (0%)	0/1 (0%)
BMI (kg/m^2^), median (IQR)	32 (26–38)	31 (26–36)	32	39
Unilateral pain, *n* (%)	10/14 (71%)	8/11 (73%)	1/1 (100%)	0/1 (0%)
Duration of pain (yr), median (IQR)	6 (4–10)	7 (4.0–10.0)	5	6
Age of onset (yr), median (IQR)	30 (25–39)	29 (23–39)	37	29
Morphine milligram equivalent (mg), median (IQR)	48 (19–136)	36 (15–120)	48	20
Number of pain medications, median (IQR)	3 (2–3)	3 (2–3)	2	3
eGFR (ml/min per 1.73 m^2^) using CKD-EPI, median (IQR)	109 (91–121)	113 (96–118)	133	80
Urea (mmol/l), median (IQR)	4.6 (4.0–5.7)	5 (4.2–5.8)	2.8	3.1
Creatinine (μmol/l), median (IQR)	64 (55–69)	63 (56–67)	31	82
ACR (<3.0 mg/mmol), *n* (%)	14/14 (100%)	11/11 (100%)	1/1 (100%)	1/1 (100%)
Proteinuria absent (urine analysis), *n* (%)	14/14 (100%)	11/11 (100%)	1/1 (100%)	1/1 (100%)
Macrohematuria, *n* (%)	11/14 (79%)	8/11 (73%)	1/1 (100%)	1/1 (100%)
Hypertension, *n* (%)	3/14 (21%)	2/11 (18%)	0/1 (0%)	0/1 (0%)
Diabetes, *n* (%)	2/14 (14%)	0/11 (0%)	1/1 (100%)	0/1 (0%)
Kidney stones, *n* (%)	8/14 (57%)	6/11 (55%)	1/1 (100%)	0/1 (0%)
Family history of kidney stones, *n* (%)	2/14 (14%)	2/11 (18%)	0/1 (0%)	0/1 (0%)
Current smoker, *n* (%)	4/14 (29%)	2/11 (18%)	1/1 (100%)	0/1 (0%)
Pain reported associated with hematuria, *n* (%)	8/14 (57%)	8/11 (73%)	0/1 (0%)	0/1 (0%)

ACR, albumin-to-creatinine ratio; BMI, body mass index; CKD-EPI, Chronic Kidney Disease Epidemiology Collaboration equation; eGFR, estimated glomerular filtration rate; IgA, immunoglobulin A nephropathy; IQR, interquartile range; kg, kilogram; l, liter; m, meter; mg, milligram; μmol, micromoles.

Biopsy for 1 patient was insufficient for electron microscopy histopathological analysis.

**Figure 2:**
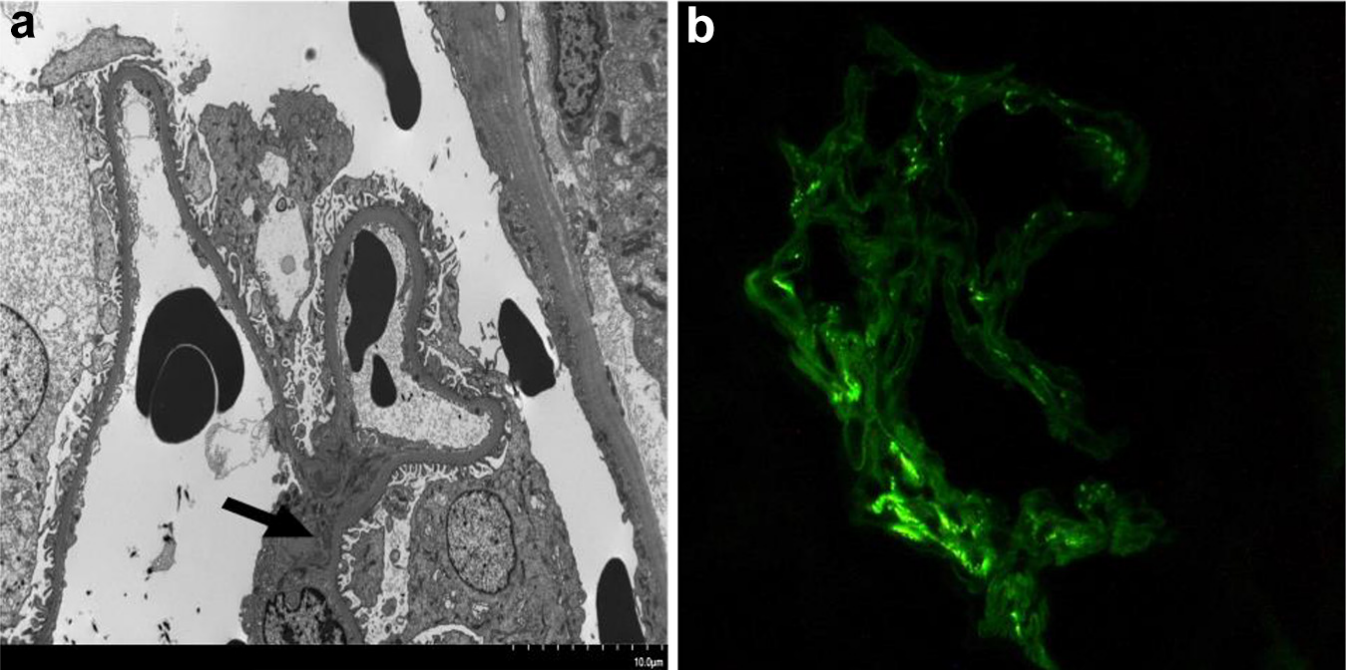
Alterations of glomerular basement membrane visible with light microscopy. Mesangial deposition of IgA in one patient. (a) IgA deposition in mesangial areas detected by electron microscopy and (b) IgA deposition detected by immunofluorescence studies in the same patient.

**Figure 3:**
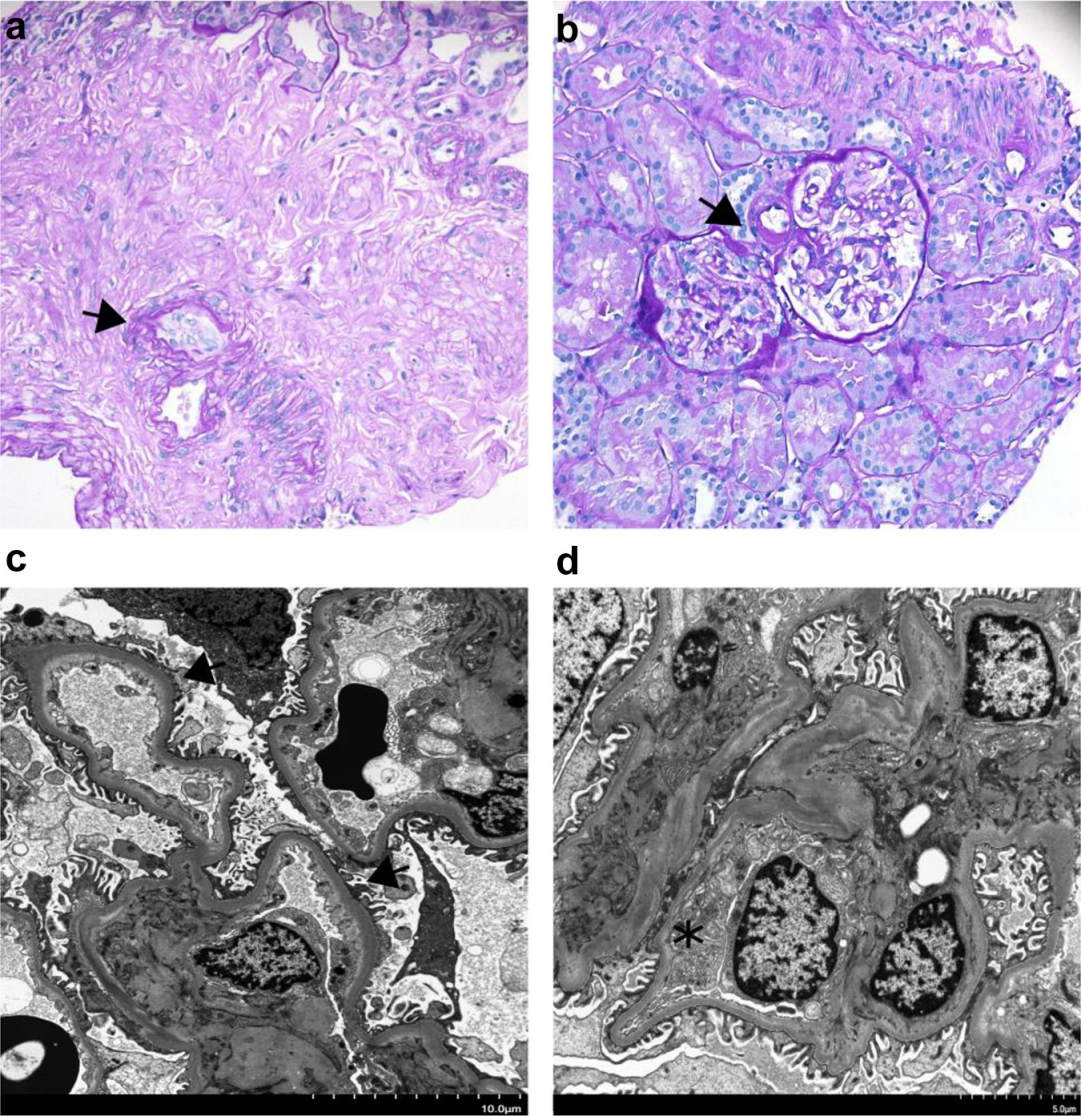
Alterations in endothelial cells. Minor alterations in Microvasculature. Subendothelial hyalinosis and enlarged endothelial cells as visualized under periodic acid–Schiff staining at 100× magnification in (a) small arteries/arterioles (arrow) and in (b) hilar arterioles (arrow). Electron microscopy images show (c) expansion of subendothelial spaces and loss of fenestration in endothelium (arrows), and (d) endothelial cell swelling partially obliterating capillary lumen.

**Figure 4:**
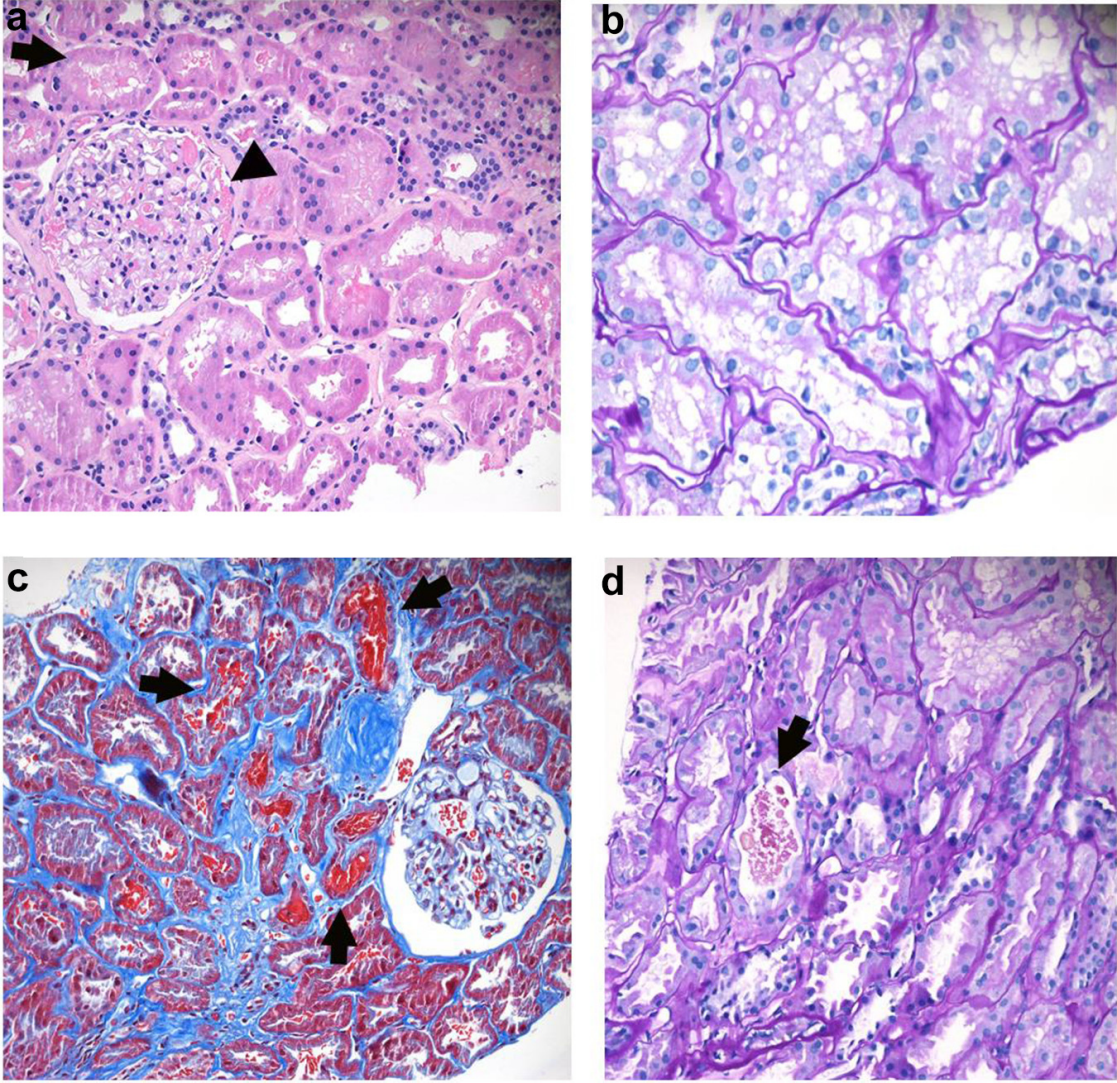
Presence of red blood cells and casts. (a) Intact and dysmorphic red blood cells are seen inside tubules (arrow) and glomerular urinary space (arrowhead)-with hematoxylin and eosin staining at 100× (b) acute tubular injury with loss of brush border, simplification, and vacuolar degeneration in epithelial cells with periodic acid–Schiff staining, 200× (c) red cell cast in many tubules with Masson Trichrome stain, 100× (d) Arrow shows dysmorphic and degenerated red blood stains with periodic acid–Schiff staining, 100×.

Histological examination of kidney biopsies from the 14 patients are found in Table 2, though inadequate sample was present in 1 biopsy and thus EM was only successful in 13 patients. None of the patients had any glomerular abnormalities, including mesangial or endocapillary hypercellularity by light microscopy. The majority (12/13) patients on complete histomorphological assessment had a normal appearing GBM, free of any structural irregularities and with average thickness within normal limits for adult women on light microscopy. One of 14 had GBM thickness above the normal cut off for women on EM. One patient had staining positive for IgA (Figure 2). Arteriolar hyalinosis was present in 4 patients and 6 had endothelial cell injury (Figure 3). Two of 4 patients with arteriolar hyalinosis were smokers, 1 had hypertension and the fourth had diabetes. Arteriosclerosis was seen in 5 patients, 4 of whom had an elevated body mass index (>25 kg/m^2^). We were unable to assess GBM thickness on EM for 1 patient because of inadequate sample. Red blood cells or red cell casts were present in the tubules or glomerular space of 10 patients (71%) (Figure 4a, b, and d). Immunofluorescence demonstrated positive C3 staining in 6 sampled arterioles. Interstitial fibrosis and tubular atrophy was present in 5 cases, whereas 3 cases exhibited evidence of tubular injury (Figure 4c).

**Table 2:** Kidney biopsy histological findings in patients with loin pain hematuria syndrome

Patient ID	1	2	3	4	5	6	7	8	9	10	11	12	13	14
Light microscopy														
Total glomeruli	17	23	32	36	28	11	8	8	5	22	45	14	27	14
Globally sclerosed glomeruli	0	5	0	4	0	1	0	1	0	1	4	0	11	2
Segmentally sclerosed glomeruli	0	0	0	0	0	0	0	0	0	0	1	0	0	0
Tubules with RBC/RBC casts	Yes	Yes	Yes	Yes	No	Yes	No	Yes	n/a	Yes	No	Yes	Yes	Yes
Tubular injury	None	None	None	Mild	Mild	Mild	None	Severe	n/a	None	None	None	None	Mod
IFTA	None	Mild	None	Mod	Mild	None	None	Mild	None	None	Mild	None	Mod	None
Arteriosclerosis	None	Mild^a^	None	None	None	None	None	No Artery	None	Mild	Mild	Mod	Mild	None
Arteriolar hyalinosis	None	None	None	Mild	None	None	None	Mod	None	Mild	None	None	None	Mild
Immunofluorescence														
C3 staining	Mild	Neg	Trace	Mod	Neg	Mod	Mild	Trace	Neg	Neg	Neg	Trace	Neg, IgA/λ +	Neg
Electron microscopy														
GBM-Avg	293	273	272	273	251	330	440	315	369	283	267	280	266	MD
GBM-Min	188	156	167	156	119	211	312	247	238	210	189	187	214	MD
GBM-Max	853	879	783	879	703	890	847	634	835	733	601	673	491	MD
GBM-range (215–395 nm)	in range	in range	in range	in range	in range	in range	Thick GBM	in range	in range	in range	in range	in range	in range	MD
% of readings <250 nm	8.7%	16.9%	13.3%	19.5%	20.5%	3.4%	0%	0.8%	0.9%	9.0%	13.4%	8.0%	5.3%	MD
Endothelial injury	None	None	Mod	None	Mild	None	Focal +	Mild	None	Mod	Severe	None	None	MD
GBM duplication	None	None	None	Focal +	None	None	None	None	None	None	None	None	None	MD
Ischemic wrinkling	Present	None	Present	None	Present	None	None	None	None	None	Present	Present	None	MD
Deposits	None	None	Fibrin Tactoid	None	None	None	None	None	None	None	None	Few +	None	MD
Genetic testing														
*COL4A3*	Neg	Neg	Neg	Neg	Neg	Neg	Neg	Neg	Neg	Neg	Neg	Neg	Neg	Neg
*COL4A4*	Neg	Neg	Neg	Neg	Neg	Neg	Neg	Neg	Neg	Neg	Neg	Neg	Neg	Neg
*COL4A5*	Neg	Neg	Neg	Neg	Neg	Neg	Neg	Neg	Neg	Neg	Neg	Neg	Neg	Neg

Arteriosclerosis, arterial intimal fibrosis; avg, average; C3, complement 3; GBM, glomerular basement membrane; IFTA, interstitial fibrosis and tubular atrophy; max, maximum; min, minimum; Neg, negative; nm, nanometer; RBC, red blood cell; TGBM, thin glomerular basement membrane.

a Myocyte vacuolization.

After successful sequencing of the *COL4A3*, *COL4A4*, and *COL4A5* genes in all 14 participants, there were no identified “pathogenic” or “likely pathogenic” variants. There was no evidence of copy number variations identifiable in the sequencing data.

## Discussion

We present kidney biopsy results and type IV collagen sequencing in 14 unrelated patients with LPHS. Eleven of 14 had a normal appearing GBM, whereas 1 of 14 had a thick GBM and 1 of 14 had evidence of IgA deposition. Red blood cells (10/14) or red cell casts (7/14) were present in multiple tubules in the majority of patients consistent with a glomerular source of bleeding. We did not identify any pathogenic *COL4A3*, *COL4A4,* or *COL4A5* variants in this cohort of patients with loin pain hematuria. Based on these results, conventional histopathology and genetic testing for type IV collagen variants failed to identify the cause of hematuria in 14 patients with LPHS.

If a biopsy is performed in a patient with isolated hematuria, examination of the GBM using EM is usually viewed as the most important component. A case series of 7 patients in 1996 reported an absence of GBM abnormalities but presence of red cells in multiple cross-sections of renal tubules, indicating that the hematuria was glomerular in origin.^6^ In contrast, Spetie *et al.*^8^ reported unusually thin (11/34) or thick (9/34) GBM in some patients but remaining 14 of the 34 patients had normal GBM thickness. They acknowledged the observed variability but suggested structural GBM abnormalities as the most common reason for hematuria and resulting interstitial edema that lead to capsule stretch as the cause of pain. In contrast, in our cohort of patients with loin pain hematuria, there was an absence of GBM thinning and only modest thickening in one patient (with diabetes), with no observation of interstitial edema that could be contributing to capsule stretch or loin pain.

Increasing access to genetic testing has improved our understanding of the spectrum of phenotypes associated with type IV collagen nephropathy and a recognition that thin basement membrane disease and Alport syndrome share a common molecular basis.^14^, ^15^, ^16^ Thin basement membrane is now viewed as a histological lesion description rather than a diagnostic entity. Classical progressive X-linked Alport syndrome occurs in males with hemizygous *COL4A5* variants^17^^,^^18^ because their one and only X chromosome carries a pathogenic variant. Homozygous variants in *COL4A3* or *COL4A4*,^19^ found on chromosome 2, can present with an autosomal recessive phenotype that is clinically similar to classical Alport syndrome. Compound heterozygous digenic inheritance has also been reported. Heterozygous *COL4A3* or *COL4A4* variants, or heterozygous *COL4A5* in women can present with a range of histological and phenotype features variably, including hematuria, proteinuria, focal segmental glomerulosclerosis, and chronic kidney disease.^14^ Genotype-phenotype correlation exists and those with more severe mutations resulting in protein truncation or impacting glycine residues result in the most severe phenotypes.^20^ These mutations lead to defects in the synthesis, assembly, and deposition of the type IV collagen protein into the GBM. More than 1000 mutations have been identified in the *COL4A3*, *COL4A4*, and *COL4A5* genes leading to a reclassification of Alport syndrome and thin basement membrane nephropathy as type IV collagen nephropathies.^14^ We hypothesized that pathogenic variants in type IV collagen could explain a proportion of patients with hematuria, but we observed zero pathogenic or likely pathogenic variants in this cohort of 14 patients with loin pain hematuria. Although we cannot exclude type IV collagen variants as a contributor to LPHS based on 14 patients, it appears unlikely to play a substantial role.

Deposition of C3, presence of arterial sclerosis, and endothelial cell injury were present in patients in this LPHS cohort but similar abnormalities have been reported in studies of healthy kidney donors. Of 102 transplant donors who underwent a kidney biopsy before transplant, 74.5% showed nonspecific IgM or C3 deposits, intimal fibrosis of small arteries (44%), interstitial fibrosis (8%), and arteriolar hyalinosis (29%).^21^ In a study of 84 donors in India, 48% had abnormal histological changes, which included glomerulosclerosis (25%), interstitial fibrosis (13%), acute tubular necrosis (5%), and focal tubular atrophy (5%).^22^ The observed changes are not likely “hidden clues” to as yet unidentified mechanisms for LPHS but rather changes that can be observed in “normal kidneys.”

It remains possible that yet unidentified defects in the endothelium and GBM lead to the leakage of red cells from the vasculature into the urinary filtrate. Red cell transit through the GBM has been associated with a transient 2.25 μm wide gaps in the endothelium and underlying basement membrane.^23^ Red cells have been shown to traverse through microscopic pores, holes, and microscopic ruptures of the GBM.^24^^,^^25^ Savige *et al.*^26^ wondered if hematuria occurred from gaps in the GBM were so small that negatively charged albumin particles were repelled from margins of the endothelium.

LPHS is likely not a distinct clinical entity but a common end point for a diverse group of underlying disease processes that manifest clinically with loin pain and hematuria. The histological changes identified in our study offer no consistent explanation for the genesis of the hematuria or pain or further basis for patient subclassification. Pathogenic type IV collagen variants are unlikely to explain a significant proportion of patients with LPHS but future genomic studies investigating additional genes that code for the GBM, proteins important for glomerular endothelial cell function, or the remainder of the exome and genome may provide additional clues. Given its rarity, genetic and genomic efforts will focus on families with multiple affected individuals.

Incapacitating, debilitating, opioid requiring pain remains a prominent feature of LPHS and its pathophysiology remains a mystery. The transmission of pain from the viscera to the brain involves a pathway that is controlled by an intricate series of genes (*N* = 420) that code for transduction, conduction, synaptic transduction, and modulation.^27^ Pain transmission pathways may be inappropriately amplified in patients with LPHS via pathogenic gain-of-function variants in pain sensing pathways or pathogenic loss-of-function variants in pain inhibiting pathways, which could also be examined.

Limitations of the current study include the small sample size conducted in a single center, which limits generalizability. However, LPHS is a rare condition and as a regional referral center, we have attempted to capture as many patients as possible. All the patients were female and majority were of European heritage. Additional studies are on-going and collaboration is welcome.

In conclusion, LPHS remains a disabling chronic condition primarily impacting women of reproductive age whose pathophysiology remains unknown. Kidney biopsy histology and sequencing of the type VI collagen genes in our cohort of 14 patients did not provide additional clues to the mechanism of hematuria or pain in patients with LPHS.

## Disclosure

This research was supported partially with a generous grant by The Hospitals of Regina Foundation. BP reports speaker and advisory fees from Bayer, Otsuka, Glaxo Smith Kline, Astra Zeneca, and Medtronic. MBL reports speaker and advisory fees from Otsuka, Bayer, Reata, and Sanofi. All authors declare no potential conflicts of interest with respect to the research, authorship, and/or publication of this article.
